# Prescriptions

**Published:** 1894-12

**Authors:** 


CHRONIC ALCOHOLISM.

℞. Tinct. capsici..........1 ounce.
Tinct. zingiberis.......1 ounce.
Tinct. valerinæ Am'on. 2 ounces.
Celerina...............2 ounces.
M. Sig.—Teaspoonful in teacupful of hot tea three or four times daily.

—*St. Louis Clinique*.

Useful in sluggish conditions of the liver:

℞. Podophyllini, gr. ¼.

Euonyminæ, gr. iss.

Ext. belladonnæ, gr. ¼.

Pil. hydrarg., gr. ii. M.

For 1 pill, to be taken at bedtime.

FOR CONTINUED SYPHILIS.

℞. Red iodide of mercury4 grains.
Iodide of potassium..4 drachms.
Tincture of iodine....2 drachms.
Distill'd wat'r, to m'ke 8 ounces.
A teaspoonful in a wineglass of water three times a day, one hour after each meal.

—*Dr. Joseph Jones*.

For gonorrhœa:

℞. Copaibæ bals., M. iv.

Ol. santali, M. v.

Ol. cinnamomi, M. i.

Ft. in capsul. No. 1.

From 6 to 12 capsules to be taken in the day, one hour after meals.

ASTHMA.

The following will be found most useful in this distressing complaint:

℞. Chloralis...............

Potassii iodidi, of each. ʒ ss.

Syrup of oranges......fl. ʒ vj.

Water.................fl. ℥ vj.

2 to 5 tablespoonfuls a day.

For incontinence of urine:

℞. Sodii benzoati, gr. xv.
Sodii salicylatis, gr. xvii.
Ext. belladonnæ, gr. xxx.
Aq. cinnamomi, q. s. ad. ℥ iv.
M. A teaspoonful to be taken four or five times a day.

Please mention Southern Medical Record.

CARDIAC STIMULANT.

℞. Tinct. strophanthi..... M. 40.

Tinct. zingib.......... ʒ j.

Syr. simp............. ʒ iss.

Aq. chlorof..q. s. ad. fl. ℥ iij.

M. S.—Two tablespoonfuls three times a day.

A good evaporating lotion:

℞. Ammonii chloridi, ℥ i.

Sp. rectificati, ℥ ii.

Sp. ætheris, ℥ i

Acid. acetic, ℥ iss.

Aq. destill., q. s. ad. ℥ xii.

Solve et M.

To be applied on lint in severe sprains, etc.

CHRONIC ALCOHOLISM.

℞. Tinct. capsici............
Tinct. zingib.........aa fl. ℥ j.
Tinct. valerian. ammo....
Tinct. gent. comp. ...aa fl. ℥ ij.

M. Sig.—Take a dessertspoonful in a teacupful of hop tea three or four times a day.—*Gerhard*

For ozæna:

℞. Acidi carbolici, gr. xxx.

Resorcin (crys.), gr. xlv.

Glycerini, ℥ iss.

Aquæ, q. s. ad. ℥ xii. M.

To be used as a spray.

INCONTINENCE OF URINE.

℞. Tinct. of belladonnæ....
Tinct. cubebæ.......aa fl. ʒ ij.
Tinct. nucis vomicæ.....
Tinct. rhois aromaticæ,
of each................fl. ʒ j.
Tinct. cascarillæ........fl. ʒ ij.

12 drops at bed-time for a child from seven to ten years.

TREATMENT FOR PAINFUL DEFECATION.

For painful defecation attending inflammatory pelvic conditions, Dr. Murray (*Norsk Magazin* for Lœgevid) recommends the following:

℞. Bismuth subnitrate, gr. iiss.
Mercurial ointment, gr. iss.
Ex. of belladonna, gr. iv. to gr. v.
Cacao butter, q. s. for 1 suppository.

Sig.—2 suppositories a day.

